# The application of an environmental performance framework for climate adaptation innovations on two nature-based adaptations

**DOI:** 10.1007/s13280-021-01571-5

**Published:** 2021-05-28

**Authors:** Jantsje M. van Loon-Steensma, Christopher Goldsworthy

**Affiliations:** 1 Water Systems and Global Change Group, Wageningen University, P.O. Box 47, 6700AA Wageningen, The Netherlands; 2 Department of Hydraulic Engineering, Delft University of Technology, P.O. Box 5048, 2600 GA Delft, The Netherlands; 3 Institute for Science, Innovation and Society, University of Oxford, 64 Banbury Road, Oxford, OX2 6PN UK

**Keywords:** Environmental impact, Flood risk infrastructure, Nature-based solutions, Sustainable design, Wadden Sea

## Abstract

In this paper, we introduce and test a framework to qualitatively assess the environmental impact of climate adaptation innovations with the ambition to facilitate the implementation of these adaptations. The framework was designed to enable continuous environmentally conscious benchmarking based on three environmental performance indicators: sustainable design, environmental impact and ecological impact. It was pilot tested by uninvolved experts and key-persons for two large-scale nature-based flood adaptation innovations in the Netherlands and discussed with environmental assessment professionals. Our findings indicate how the inclusion of our framework helps to identify important knowledge gaps regarding environmental co-benefits and trade-offs, and can be beneficial to both those developing the innovation and the local authorities charged with assessing the suitability of innovations. We conclude by noting how the incorporation of environmental impact assessment from the design stage of adaptations could supplement existing environmental assessment regulations pre-empting concerns rather than reacting to them.

## Introduction

The increasing frequency and intensity of climate-related extreme and devastating events calls for major climate adaptation efforts and innovative solutions (e.g. EEA 2012; IPCC 2012; Kovats et al. 2014; Noble et al. 2014). Many countries have initiated policy programmes and comprehensive research programmes to develop national and regional adaptation strategies and adaptation measures (Abeysinghe et al. 2017; McEvoy et al. 2021), and many municipalities have established local programmes and stimulated stakeholder groups to develop and implement local (mostly urban) adaptation measures. These adaptation measures may be structural (e.g. engineered flood protection works, shelters, green roofs, retention areas, drainage or irrigation infrastructure), social (e.g. awareness raising, vulnerability mapping, monitoring) or institutional (e.g. insurance schemes, land zoning laws) (Noble et al. 2014). In Europe, the development of adaptation strategies and new solutions is since 2013 supported by a formal EU strategy on adaptation to climate change (EC 2013a, b). This includes major funds dedicated to develop and implement innovative ways to respond to climate challenges (www.ec.europa.eu/clima/policies, www.ec.europa.eu/programmes/horizon2020). As a result, researchers and innovators have been developing numerous climate adaptation innovations (see e.g. www.climate-adapt.eea.europa.eu; www.brigaid.eu).

Despite ample attention for the increasing climate risks and all efforts to develop adaptation strategies and stimulate innovation, the application and implementation of adaptation innovations is still modest (Kovats et al. 2014). This has been attributed to a lack of communication and common interest between adaptation innovators (researchers and companies) and end-users (ranging from governmental institutions to households) (www.brigaid.eu). Increased efforts have been supporting the development and implementation of adaptation innovations. For instance, with guidance on readiness and advice on steps to advance technological readiness to a more mature stage (www.brigaid.eu). As a first step, Lendering et al. (2018) developed a framework to assess an innovation’s technical performance to reduce the hazard potential (e.g. by reducing the likelihood of the hazard or its intensity via infrastructure) or the vulnerability of the people and assets in the area at risk (e.g. by providing information, spatial planning, emergency planning, capacity building) (EEA 2012; Klijn et al. 2015). Their assessment is based on four technical performance indicators: effectiveness, durability, reliability and costs (Lendering et al. 2018). However, climate change is a complex, multifaceted problem, and uptake of innovations does not only depend on their technical readiness and performance compared to traditional solutions, but also on their social readiness (Wilbanks et al. 2007), and their impact on socio-economic sectors (Hallegatte et al. 2011). Bellamy (2019) reflected on the four common dimensions of social readiness of technologies: knowledge of technology, scope of technological projects, impacts of technology, and trust in the control of technology, and argued that including these dimensions in the search for suitable adaptations could help to find socially acceptable adaptation technologies.

So far, undesirable environmental impacts of climate adaptations receive less attention than technical, social and economic perspectives (Enríquez-de-Salamanca et al. 2017), while engineered structural solutions may affect the quality of the environment, biodiversity, and landscape values. For instance, closure dams designed to protect the estuarine coast by closing off the river mouth or estuary may be very effective to reduce flood risks in the delta, but they also form a physical barrier in the estuarine system that will hamper species migration and may disturb the ecological valuable gradient in saline habitats resulting in loss of biodiversity or a decrease in water quality (Linham and Nicholls 2010). On the other hand, there are many international and national agreements and laws in place to protect, preserve and improve the environment (e.g. acts on Water Quality, Air Pollution, Waste Disposal). If an adaptation measure is expected to have significant effects on its environment (e.g. the construction of a dike or a water retention area), or that implementation will require a substantial amount of space (that is for instance, currently designated as nature area), then an Environmental Impact Assessment (EIA) is often legally required. An EIA ensures that the environmental implications of measures are taken into account before the decisions on implementation are made, by comparing their impact with some alternative solutions (SEA Directive Environmental Assessment 2001/42/EC; EIA Directive 2011/92/EU). For smaller measures, as is the case for many (urban) adaptation innovations, or for measures with no foreseen significant effects on the environment, an EIA may not be obliged by legislation. Innovations with significant perceived co-benefits for e.g. nature or landscape values, or reduced trade-offs on the environment compared to conventional solutions may form ‘no-regret solutions’ and may suit policies on sustainability, green growth, or nature conservation. However, Lach, Rayner and Ingram (2005) and Rayner (2012) make it clear that even in situations in which ‘no-regret solutions’ are deployed there can be unforeseen or hidden environmental or socio-economic costs.

There are also adaptation innovations that emphasize their positive impact on the environment: Nature-based solutions (NBS). NBS are inspired and supported by nature, and often result in conservation or even in the development of nature and improved environmental quality (Cohen-Shacham et al. 2016). Furthermore, NBS may simultaneous provide social and economic co-benefits (EC 2015; Faivre et al. 2017; Kabisch et al. 2016, 2017; Raymond et al. 2017). While NBS are on the one hand in line with concepts such as ‘natural capital’ and ‘ecosystem services’, which emphasize the important services provided by nature and focus on nature conservation and restoration (Costanza et al. 1997; MEA 2005, see also Nesshöver et al. 2017), NBS could also been seen as eco-, or green infrastructure innovations that fit in a technical change towards ‘sustainable development’ (e.g. Brundtland et al. 1987; OECD 2009; Smith et al. 2010; De Vriend et al. 2015). During the past few years, various studies (including conceptual frameworks) have provided insights into the relationship between sustainability and NBS (Raymond et al. 2017), the multifaceted and multifunctional character of NBS, and its implications for science, policy and practices (e.g. Eggermont et al. 2015; Kabisch et al. 2016, 2017; Nesshöver et al. 2017; Kalantari et al. 2018; Seddon et al. 2020). In addition, substantial work is being done on building up an evidence and knowledge base by collecting cases (e.g. www.naturebasedsolutionsevidence.org), identifying indicators for assessing the effectiveness of NBS (e.g. Kabisch et al. 2016, Raymond et al. 2017), and to conceptualize, map or model the economic, social and environmental benefits (e.g. Lafortezza and Sanesi 2019). It is also increasingly recognized that NBS, like all other innovations, may result in some trade-offs additional to their intended performance and foreseen co-benefits (van Loon-Steensma and Vellinga 2013; Raymond et al. 2017; Turkelboom et al. 2018; Seddon et al. 2020).

Insights into co-benefits and trade-offs on nature and the environment as well as compliance with other policy fields could facilitate the benchmarking of innovations and support the decision process on implementation. In the face of uncertainty about pace and impacts of climate change and socio-economic developments (Hallegatte 2009), there is a particular interest in ‘no-regret’ solutions. Furthermore, insights into environmental impacts may help to modify the design of adaptation innovations early on towards more sustainable solutions and to avoid innovations that become prematurely locked-in, and so precluding the consideration of others that may be better suited to tackling the issue at hand. We therefore argue for the explicit consideration of environmental impact in the design process of all climate adaptation innovations, including NBS, in addition to existing technical performance indicators.

The aim of this paper is to introduce a multi-dimensional environmental performance framework for adaptation innovations to identify compliance with other policy fields, co-benefits and trade-offs early on in the design process. We apply this framework to consider the environmental performance of two nature-based solutions in the Dutch Wadden Sea. Research questions are: (1) what are relevant and manageable dimensions and criteria for an environmental assessment of climate adaptation innovations? (2) How can environmental impacts be integrated within the design process of climate adaptation innovations? (3) What are the potential environmental impacts arising from the application of the environmental performance framework on two large-scale nature-based adaptations? and (4) What is the value of an environmental performance framework and how to implement it?

## Materials and methods

This section first explains our conceptual approach, how we identified indicators and variables, and how our Environmental Performance Framework can support the design process of adaptation innovations. Next, it explains how we pilot tested our framework and reflected on its value. Finally, it introduces the two large-scale nature-based climate adaptation innovations.

### An indicator-based framework to assess the environmental impact of climate adaptation innovations

#### Conceptual approach

**Fig. 1:**
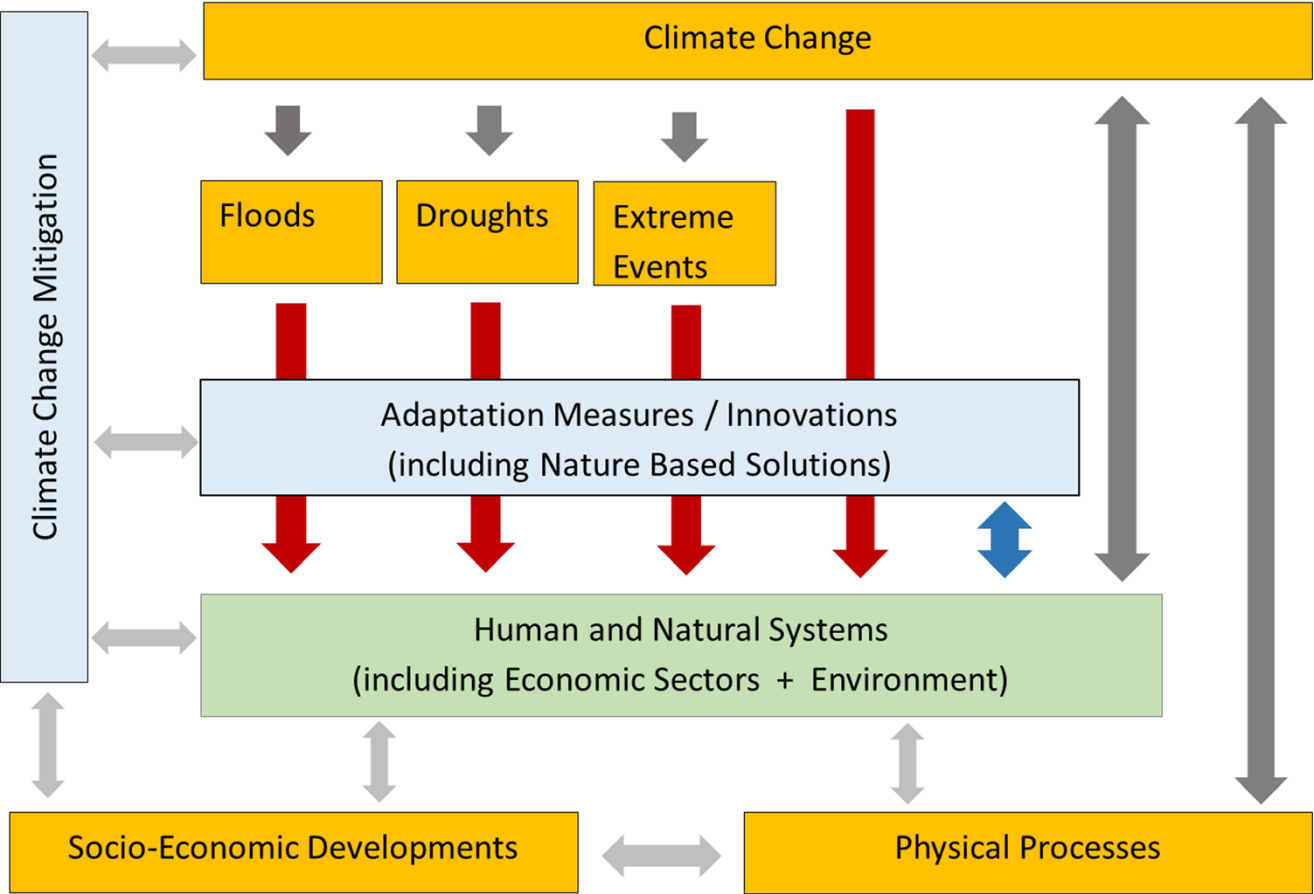
Schematic interaction between adaptation measures and human and natural systems. This figure illustrates how adaptation measures mitigate climate change impacts and climate-related disasters (floods, droughts and extreme events) (red arrows). Additional there may be impacts from adaptation measures on human and natural systems (blue arrow)

Climate change will increasingly and unavoidably affect our environment through rising temperatures and the associated changes in precipitation patterns and sea level rise (IPPC 2007, 2014). While various measures are known to prepare for and respond to these changes, the fact that these will also be part of the climate-environmental system with dynamic relations and multiple feedbacks is often not fully appreciated (Enríquez-de-Salamanca et al. 2017). Consequently, there is a tendency to overlook potentially adverse side-effects of these measures. Furthermore, climate adaptation measures are taken in a context where non-climate-related threats or impacts from geo-physical processes and socio-economic developments (like urbanization, population growth and changes in policy) are also highly relevant (e.g. Smith 1993, IPPC 2007, 2014; EEA 2012). Hence, we aim to design an approach which identifies both positive and negative side-effects of potential climate adaptation measures and at the same time prioritizes measures with mutual benefits for different sub-systems (or actors) and reconciliation of potential conflicts. Figure 1 highlights our systems view that underlies our approach to identify and select ideal climate adaptation measures.

#### Identification of manageable and meaningful criteria

Our exploring approach started with discussions with some 30 international innovators and end-users (of mostly small-scale urban) adaptation innovations. An important outcome of these initial consultations was that the environmental assessment of adaptation innovations should be distinctive from an EIA (EIA Directive 2011/92/EU). The innovators and end-users had a strong preference for a (qualitative) method that they could initially apply themselves. They raised that the tool should cover different approaches, like sustainability, green economy, circular economy, ecosystem services, environmental risks, and nature restoration and ecosystem conservation. Another outcome was that the tool should take into account temporal and spatial aspects. For instance, structural/physical adaptation innovations may impact the environment during their construction, implementation or operational phase. This impact may be positive or negative, direct (those caused by the preparation, construction, or operation of an innovation at a particular location) or indirect (those that spatially or temporally distant from the innovation), temporary—short or long term—or permanent, or reversible with some additional efforts. Many of the impacts are uncertain or dependent on local factors, like the ecosystems present and their current quality, land use, soil conditions, and characteristics of the water system. Furthermore, the environmental impacts of adaptation innovations that will be temporarily operated, may also depend on the duration and severity of a hazard event together with the exposure, vulnerability and resilience of the human and natural system at stake.

Therefore, we have drawn up an assessment tool, that pragmatically integrates and operationalizes various sustainability-related concepts and approaches, and contains indicators for water, soil and air and for nature. The latter are based on environmental related agreements and legislation (e.g. acts on Water Quality, Air Pollution, Waste Disposal, Convention on Biological Diversity (CPB)), experiences with evaluation methods and frameworks for adaptation measures (such as EIA, cost-benefits analyses for the Dutch Delta Programme (see e.g. Lamberigts et al. 2012)) and literature on the wide range on indicators (e.g. Raymond et al. 2017; von Thenen et al. 2020). In line with CPB and the United Nations Environmental Programme (UNEP) we included the number of (target) species as a measure of ecological quality.

Table 1 presents the criteria in our Environmental Performance Framework (EPF) on (1) the sustainability of the adaptation’s design and its contribution to sustainable development, (2) the quality of the environment, and (3) the quality of ecological systems. A guidance document helps innovators through interpreting the results of their self-assessment and explains that many assessment questions serve to identify potential environmental concerns to discuss with stakeholders and end-users and might be addressed early in the design process.

**Table 1:** Environmental Performance Framework (EPF) to evaluate an adaptation innovation’s impact related to the reference situation

Environmental performance			
Indicators			Description
A	Sustainable design		
	A1	Nature-based	Does the innovation deliberately use ecosystems and their services, or mimic or preserve natural processes? (A) Yes (B) No, and the innovation may hinder natural processes or services provided by ecosystems, (C) No, but the innovation does not affect the ecosystems present nor natural processes
	A2	Areal footprint	How does the change in footprint (area) required for implementation on-site compare to conventional measures or the present situation? (A) Increase space required (B) Decrease space required (C) No Impact on space required
	A3	Carbon footprint	How does the construction or operation of the innovation affect the quantity of greenhouse gases in the environment (e.g. as CO_2_ or CH_4_)? (A) Increase (B) Decrease (C) No Impact
	A4	Circular economy	Is the innovation made from recycled or recyclable materials? (A) Yes (B) No, it is made of non-recyclable materials (C) Partly
	A5	Ecosystem services	Does the innovation include specific design features or components which preserve or enhance ecosystem services? (A) Yes (B) No, and the innovation may hinder natural processes or services provided by ecosystems (C) No, but the innovation does not affect the ecosystems present nor natural processes
B	Environmental impact		
	B1	Water quality	How does the innovation impact the quality of surface water? (A) Improve (B) Worsen (C) No Impact
	B2	Water quantity	How does the innovation impact the quantity of available surface water? (A) Increase (B) Decrease (C) No Impact
	B3	Groundwater quality	How does the innovation impact the quality of ground water? (A) Improve (B) Worsen (C) No Impact
	B4	Groundwater quantity	How does the innovation impact the quantity of available ground water? (A) Increase (B) Decrease (C) No Impact
	B5	Seawater quality	How does the innovation impact the quality of the sea water? (A) Improve (B) Worsen (C) No Impact
	B6	Soil quality	How does the innovation impact soil quality? (A) Improve (B) Worsen (C) No Impact
	B7	Air quality	How does the innovation impact air quality? (A) Improve (B) Worsen (C) No Impact
	B8	Debris	Does the implementation (or construction) of the innovation generate debris? (A) Yes (B) Debris can even be stored or captured by the innovation (C) No
	B9	Noise	Does the implementation (or construction) of the innovation generate noise or vibration? (A) Yes (B) It even dampens noise (C) No
	B10	Landscape quality	How does the innovation impact landscape quality? (A) Improve (B) Worsen (C) No Impact
C	Ecological impact		
	C1	Area protected nature	How does the innovation impact the spatial extent of protected nature area? (A) Increase (B) Decrease (C) No Impact
	C2	Quality of protected habitats	How does the innovation impact the quality of protected habitats? (A) Improve (B) Worsen (C) No Impact
	C3	Protected species	How does the innovation impact the number protected species (e.g. birds, vegetation, fish, mammals)? (A) Increase (B) Decrease (C) No Impact
	C4	Area non-protected nature	How does the innovation impact the spatial extent of non-protected nature area? (A) Increase (B) Decrease (C) No Impact
	C5	Quality of non-protected habitats	How does the innovation impact the quality of non-protected habitats? (A) Improve (B) Worsen (C) No Impact
	C6	Non-protected species	How does the innovation impact the number non-protected species (e.g. birds, vegetation, fish, mammals)? (A) Increase (B) Decrease (C) No Impact

#### Supportive role of the EPF in the design process of adaptation innovations

The indicators are meant to evaluate whether the innovation may have foreseen impacts on the environment relative to the present situation (i.e. reference situation). The foreseen difference in effects of the innovation with the reference situation are qualitatively ranked on a three-point scale, varying from negative effects (− 1), no effects (0), to positive effects (+ 1), or may not be applicable. A summary of the results provides the innovator with an advice on the environmental performance of the design.

**Fig. 2:**
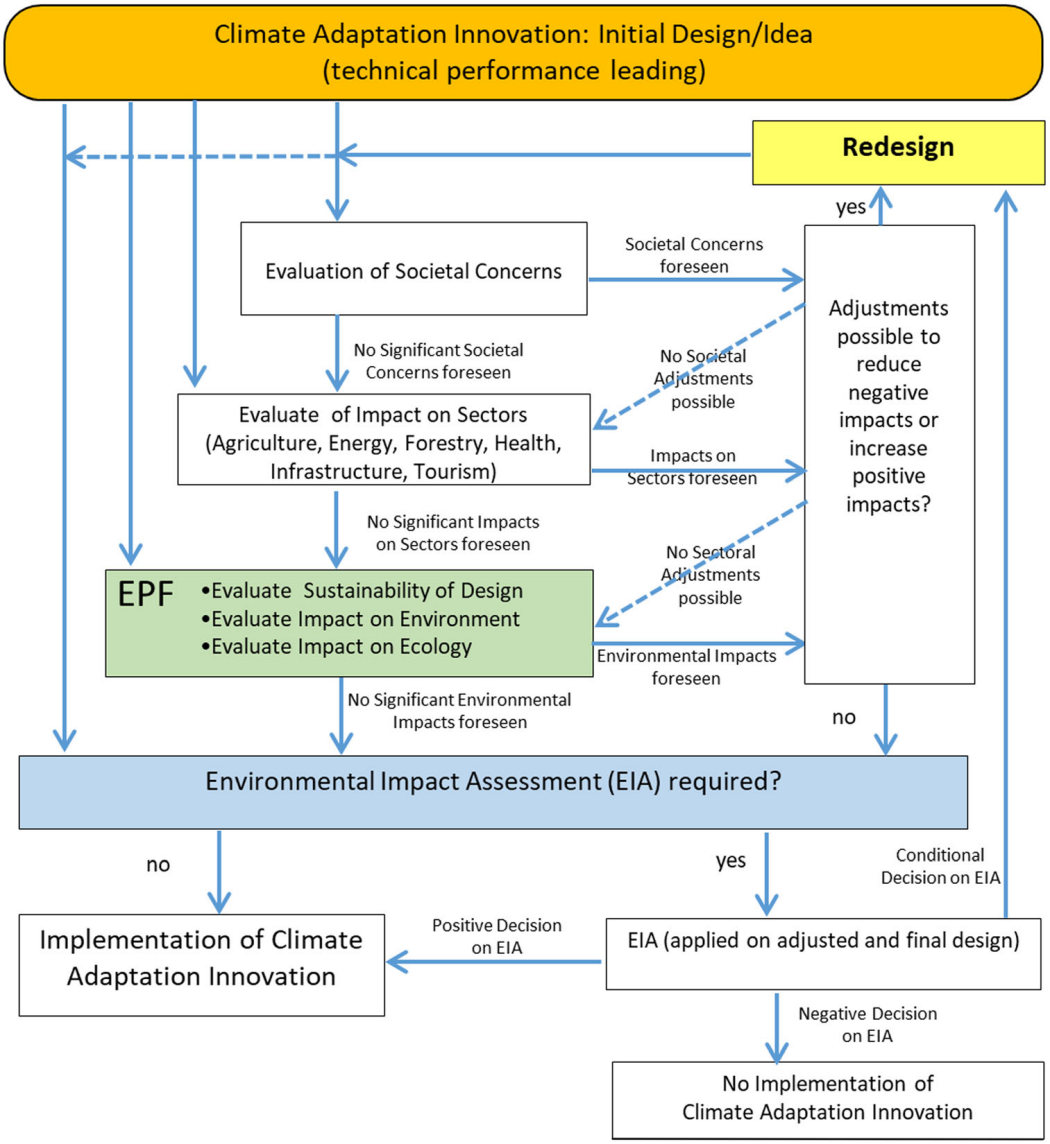
Overview of the iterative process resulting from a broad impact assessment of a climate adaptation innovation (with our EPF in the green box)

Although it might be possible for most EPF indicators to collect site-specific detailed quantitative information, our framework is designed to be initially used in a qualitative way by users who are not environmental experts. It is meant to work as an impact sieve: if no impact is foreseen, then implementation of the innovation may not meet legal or societal concern forthcoming from environmental issues, while innovations with foreseen negative impacts on the environment may require adjustments in their design, and may expect legal and societal concern and can be subject to an EIA (Fig. 2). However, whether or not EIA is required, our environmental performance framework draws attention to environmental co-benefits and trade-offs that are not addressed in an EIA, but may be important for the implementation phase. Furthermore, based on the outcomes of the assessment, experts familiar with local site-specific conditions can be asked to make quantitative analyses or as a next step (which is not yet included in our approach) to relate the impacts to the ‘*business*-*as*-*usual*’ approach (i.e. autonomous development within current policy and baseline climate change scenario) over the short and long term.

Note that next to technical performance (Lendering et al. 2018) and environmental performance (this paper), also societal concern and impact on socio-economic sectors are important issues (Bellamy 2019) that should be taken into account in the design process (Fig. 2).

### Application of the environmental performance framework

Having developed a framework to assess *ex*-*ante* the environmental performance of adaptation innovations, the next step is to apply this framework and to explore its value in the implementation process. The process from designing an adaptation innovation (after a carefully problem analysis and identification of appropriate measures, preferably in a participative way with a range of stakeholders) to real implementation normally takes a long time (e.g. for the Wide Green Dike some 10 years). Therefore, we applied our framework on two large-scale nature-based flood adaptation innovations in the Netherlands that are currently being carried out and monitored and used a mixed methods approach to explore its value to identify environmental impacts and to facilitate implementation of nature-based innovations.

First, we conducted a pilot expert interdisciplinary session with six graduate students, specializing in the field of climate adaptation. They were not yet familiar with both adaptation innovations. After a brief introduction of both innovations, we asked the group to deliberate on and jointly rank the potential impact of these innovations referring to the criteria outlined in the EPF, as well as to reflect on the value of such an assessment. To understand the effect of our EPF on their perception of nature-based flood adaptation innovations (viz. Bellamy et al. 2019) we asked their feedback on the relevance and completeness of the criteria to identify the potential impacts of these two nature-based innovations, as well as on its value to explore different views and potential controversies, initiate discussion, and learn from different views about the environmental impact of nature-based innovations.

Second, we asked the project leader of each adaptation innovation to rank the impact of the innovation and to elaborate on the environmental issues related to the implementation of the adaptation innovation. Furthermore, we invited them to reflect in hindsight on the value of an environmental performance framework for the implementation process.

Third, we invited four EIA experts (from a consultancy firm, dredging contractor, regional authority, and national EIA commission), to reflect on the applicability of our environmental performance framework and how such a framework could be implemented in a real-world context.

### Two large-scale nature-based adaptation innovations

**Fig. 3:**
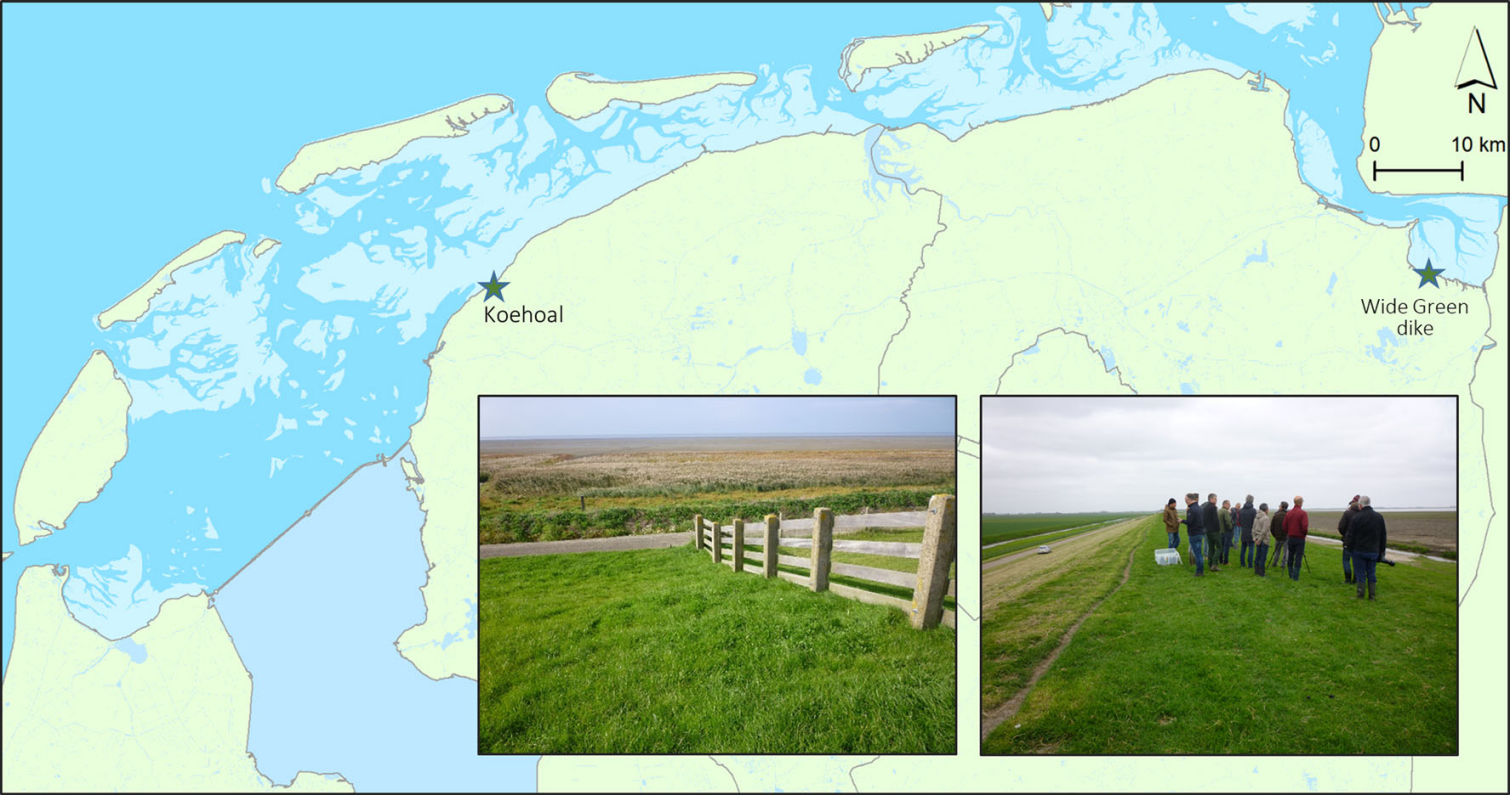
Locations and pictures of the Mud Motor Koehoal (left) and Wide Green Dike (right) nature-based adaptation innovations along the Wadden Sea coast

Our two nature-based adaptation innovations (1) Mud Motor Koehoal, and (2) Cyclic clay mining for a Wide Green Dike, are situated along the Wadden Sea coast of the low-lying northern part of the Netherlands (Fig. 3). This region is protected against flooding from the Wadden Sea by dikes. The Wadden Sea is a shallow sea, marked by barrier islands, sand and mud flats and coastal marshes (Reise et al. 2010). Due to its outstanding nature values the Wadden Sea has been listed as a UNESCO World Heritage site since 2009 (CWSS 2008; UNESCO 2009), and is protected by both national and international regulations for nature conservation. In 2010 a process commenced to search for suitable and sustainable adaptation strategies under future sea level rise (van Alphen 2016). Hybrid solutions that include the natural and semi-natural salt marshes along the Wadden Sea in dike design were seen as especially promising (Delta Programme 2014). These salt marshes function as a natural flood defence in front of the dike by damping incoming waves and reduce wave energy via friction with vegetation and the marsh surface (e.g. Anderson and Smith 2014). This has positive implications for the required dike dimensions (in particular slope and height) and the need for slope and toe protection structures (e.g. hard revetments and rocks) (Van Loon-Steensma 2015). Furthermore, natural and semi-natural salt marshes provide valuable habitat for characteristic salt-marsh vegetation (see e.g. Adam 1990), birds, fish and several invertebrate species (Bakker et al. 2005). Both our adaptations are large-scale nature-based flood protection innovations and focus on the development of salt-marsh foreshores and natural processes in view of future flood protection.

#### Mud Motor Koehoal

The development and conservation of the majority of the salt marshes along the Wadden Sea mainland coast is facilitated by reclamation works like low brushwood dams and drainage ditches (Van Loon-Steensma 2015); however, due to local favourable conditions a small strip of salt marshes has naturally developed at Koehoal site (Fig. 3).This provided an opportunity to test the potential of an innovative approach to enhance salt-marsh development by sediment management. Dredged sediment of the harbour of Harlingen was over two winter seasons not deposited on the usual spot near the harbour, but transported by vessels to a location further away to let natural processes spread the sediment to nearby Koehoal salt-marsh (Baptist et al. 2019). In this way salt-marsh development was stimulated while maintaining—or even enhancing—the ecological valuable gradients in habitats. Furthermore, transport further away to a tidal channel, dispersion with the tidal flow towards nearby mudflats and salt marshes, and capture of the dredged sediment in the salt-marsh would also prevent the recirculation of the sediment to the harbour, and thus reduce maintenance dredging (www.ecoshape.org). The Mud Motor pilot was initiated by the ‘EcoShape’ consortium of private parties, government organizations, research institutes and NGOs, and funded by the nature development programme of ‘*Waddenfonds*’ (a foundation set up in 2006 with revenues from natural gas extraction from the Wadden Sea, to compensate for the negative effects of gas extraction on Wadden Sea nature).

#### The wide green Dollard-dike

At the time of this writing, preparations have commenced to transform a section of some 1 km of the dike along the Ems-Dollard estuary (in 2021–2023) into a ‘Wide Green Dike’. Wide green dikes use only natural materials, such as clay covered with grass, have a mildly sloping seaward face that merges smoothly into the adjacent salt-marsh, and include a salt-marsh foreshore in their design (Van Loon-Steensma and Schelfhout 2017). Because the salt-marsh foreshore and the gentle seaward slope reduces wave impact, the grass-covered clay layer is sufficient to protect the dike against erosion during extreme events. Therefore, no stone or asphalt revetment is required (Van Loon-Steensma and Schelfhout 2017).

In the period prior to this adjustment of the traditional dike into the innovative wide and green dike, possibilities to apply local mined clay are explored in two experimental pilot locations. Ripening and drying of clay excavated from a semi-natural salt-marsh and a polder under tidal influence (polder Breebaart), and of dredged Ems-Dollard silt is intensively researched and evaluated as building material for the new Wide Green Dike. Removing silt from the Ems-Dollard estuary is expected to improve the water quality, and thus the ecological quality of the estuary. Water quality in the estuary, vegetation development, geomorphological development of the salt-marsh, as well as sediment deposition in the excavation pit are meticulously monitored. The Wide Green Dike and the clay mining pilot were initiated by the local water board, and financing came partly from *Waddenfonds*, because of the green character of these innovations (Van Loon-Steensma and Vellinga 2019).

## Results

### Environmental performance of ‘Mud Motor’ and ‘Wide Green Dike’

The joint ranking of the evaluation criteria in our EPF led to lively discussions among the expert team, in which differences in knowledge, as well as in viewpoints emerged. Some assessment criteria initiated discussions about definitions or approaches. The experts found it, for instance, difficult to determine whether the permanent re-use of dredging sludge is a measure that fits in with a circular economy. Furthermore, they did not know how to value newly created habits versus the value of the existing habitats in Natura2000 protected environment, or how to value the conservation of habitats on the expense of natural (erosion) processes.

The project leader of the Mud Motor raised that delineating the extent of impacts is very difficult, and often does not overlap for different processes and effects caused by the intervention. For example, only the impact of the Mud Motor on the marine and coastal environment was considered, but new salt marshes along the coast can also influence the fresh water availability in the hinterland via seepage. The impact of this salinization in the hinterland is context-depended: negative for agriculture, but positive for brackish nature. Furthermore, there might be shifts in impacts over time. The Mud Motor, for instance, had a negative impact in the short term due to increased turbidity and temporary noise, but in the long term it would improve the quality of the seawater by capturing sediment. The clay mining pit, on the other hand, implies a temporal shift from a mature salt-marsh habitat towards more biodiverse pioneer salt-marsh zone, but sediment accretion will steer vegetation development towards the mature salt-marsh in some 20 years. This implies that net impact will be neutral in the long term.

Tables 2 and 3 present the results of the assessment by the expert team and the project leaders for both innovations, and also summarizes their remarks on the application of the EPF indicators. Interestingly, for the Mud Motor the assessments by the expert team and project leader are more consistent than for the Wide Green Dike (see Tables 2 and 3). Regarding the environmental quality of the Wide Green Dike there was a difference of 6 (based on the assessment of 10 sub criteria). In contrast to the project leader, the expert team foresaw issues with the quality and quantity of freshwater in the hinterland. In reality, in response to concerns of landowners, a monitor programme has been started and agreements were made about measures to prevent problems with fresh water availability. Furthermore, the expert team *ex*-*ante* perceived several ecological co-benefits of the Wide Green Dike-claypit system, while the project leader considered the ecological impact neutral because the temporal disturbance of the protected Natura2000 habitats would only result in a temporal shift of habitats.

**Table 2:** Results of the assessment of Mud Motor with the EPF by an expert team (ET) and the project leader (PL) and a summary of their remarks on application of the indicators

Environmental performance			Mud Motor		Remarks
			ET	PL	
Indicators			Score		
A	Sustainable design				
	A1	Nature-based	A (1)	A (1)	
	A2	Areal footprint	A (− 1)		ET raised that including a salt-marsh foreshore does require more space than a traditional solution, but in this case this might not negative. PL raised the issue that the increase of a salt-marsh area in fact implies a shift towards a for safety useful habitat, and prevents a traditional dike-reinforcement (which normally needs space).
	A3	Carbon footprint	B (1)	?	Although it is clear that the development of salt-marsh captures CO_2_ (ET), the sailing of the transport vessels results in increase of greenhouse gas emission (PL). The net effect is not yet known.
	A4	Circular economy	A (1)	A (1)	This question raised in the ET some discussion about the re-use of mud when it was actually transformed into a nature protected salt-marsh.
	A5	Ecosystem services	A (1)	A (1)	Although NBS are based on the application of certain ecosystem services, they may have trade-offs for other ecosystem services, such as intrinsic value (ET and PL).
*Subscore (range* − *5 to 5)*			3	3	
B	Environmental impact				
	B1	Water quality	C (0)		PL raised that spatial scale is important. Development of salt-mash foreshore may affect seepage and subsequently the salinity of the inland freshwater. The impact is determined by land use in the hinterland (agricultural or e.g. brackish nature).
	B2	Water quantity	C (0)	C (0)	
	B3	Groundwater quality	A (1)	A (1)	See B1. A salt-marsh foreshore affects groundwater quality in the hinterland near the dike by reducing seepage (and thus salinization).
	B4	Groundwater quantity	C (0)	A (1)	It does certainly impact the quantity, but this was not monitored (PL).
	B5	Seawater quality	A (1)		On the short term it has a negative impact because of increased turbidity, but on the long term it improves the quality of the seawater by capturing sediment (ET).
	B6	Soil quality	B (− 1)	C (0)	In the ET it was raised that dredging material of the harbour could contain contaminations.
	B7	Air quality	C (0)	B (− 1)	PL raised that dredging vessels do produce air contaminants (like NO_x_).
	B8	Debris	C (0)	C (0)	
	B9	Noise	C (0)	A (1)	PL raised that dredging and sailing do temporary produce noise (likewise the normal dredging process of the harbour). However, other areas are now impacted by noise production.
	B10	Landscape quality	A (1)	A (1)	
*Subscore (range* − *10 to 10)*			2	3	
C	Ecological Impact				
	C1	Area protected nature	A (1)	C (0)	PL raised that the area of protected nature is laid down in legislation, and will not change.
	C2	Quality of protected habitats	A (1)	A (1)	PL mentioned that there was a lot of discussion about the potential impact of the Mud Motor on the quality and the legal conservation of protected habitat.
	C3	Protected species	A (1)	A (1)	
	C4	Area non-protected nature	A (1)	C (0)	PL raised that there is a shift in habitats, but no change in areal extent.
	C5	Quality of non-protected habitats	A (1)	A (1)	
	C6	Non-protected species	A (1)	A (1)	PL mentioned that the Mud Motor does certainly affect non-protected species, but at forehand the extent was not clear. Therefore, monitoring was required.
*Subscore (range* -*6 to 6)*			6	4

**Table 3:** Results of the assessment of Wide Green Dike with the EPF by an expert team (ET) and the project leader (PL) and a summary of their remarks on application of the indicators

Environmental performance			Cyclical clay mining for a Wide Green Dike		
			ET	PL	Remarks
Indicators			Score		
A	Sustainable design				
	A1	Nature-based	A (1)	A (1)	
	A2	Areal footprint	A (− 1)	A (− 1)	ET raised that creating a cyclical system that uses salt-marshes to mine clay does require more space than a traditional solution. PL explained that the clay mining pit implies a temporal shift in habitat (from salt-marsh via a pond to pioneer salt-marsh zone), so the long-term net impact is not negative.
	A3	Carbon footprint	C (0)	B (1)	According to PL, sustainability is an important principle for cyclical clay mining for a Wide Green Dike, and it is expected (though not monitored) that it reduces greenhouse gas emission compared to a traditional reinforcement (application of asphalt or transport of high quality clay).
	A4	Circular economy	A (1)	A (1)	This question raised in the ET some discussion about the possibilities to re-use the mud when it was actually used to reinforce the dike. PL stressed that clay from a dike could be re-used for a new dike, or for agriculture (to improve soil quality, or the heighten agricultural lands).
	A5	Ecosystem services	A (1)	A (1)	PL explained that integration of nature and natural processes in the design is a deliberate ambition.
*Subscore (range* − *5 to 5)*			2	3	
B	Environmental impact				
	B1	Water quality	B (− 1)	C (0)	PL: monitoring of the landward drainage ditch is planned to verify the foreseen nil impact on the water quality in the hinterland.
	B2	Water quantity	B (− 1)	C (0)	ET raised that seepage of saline water in hinterland might increase.
	B3	Groundwater quality	B (− 1)	C (0)	PL: an agreement with landowners was signed to prevent (and monitor) water quantity issues in the hinterland.
	B4	Groundwater quantity	B (− 1)	C (0)	ET raised that seepage of saline water in hinterland might increase.
	B5	Seawater quality	A (1)	A (1)	PL: it is foreseen that cyclic clay mining would improve the water quality in the Ems-Dollard Estuary by removing surplus of sediment. When the pilot would expand, then the impact on the seawater quality will be measured.
	B6	Soil quality	B (− 1)	C (0)	
	B7	Air quality	C (0)	C (0)	
	B8	Debris	C (0)	C (0)	
	B9	Noise	C (0)	C (0)	
	B10	Landscape quality	C (0)	A (1)	PL: Although there will be a temporary visible impact of the sediment depot, on the long term both the wide green dike and the excavation pond will increase the quality of the landscape (certainly compared to a traditional reinforcement).
*Subscore (range* − *10 to 10)*			− 4	2	
C	Ecological impact				
	C1	Area protected nature	C (0)	B (− 1)	PL: there will be some negative impact due to the dike, but the excavation pond will only result in a shift of habitat.
	C2	Quality of protected habitats	A (1)		PL: the answer is difficult, because there will be a shift in habitat. Implementation of the wide green dike will prevent the application of asphalt and stone revetment.
	C3	Protected species	A (1)	A (1)	PL: the island within the excavation pit will prevent predation of birds by e.g. foxes; the number of birds will be monitored.
	C4	Area non-protected nature	C (0)		
	C5	Quality of non-protected habitats	A (1)		PL: not applicable, because the area is appointed as Natura 2000 site.
	C6	Non-protected species	A (1)	C (0)	
*Subscore (range* − *6 to 6)*			4	0	

### Experiences with the application of the Environmental Performance Framework on the two NBSs

According to the experts and both project leaders our EPF encompasses many important issues relevant for the design and implementation of adaptation innovations, and helps to explicitly describe the impacts on a broad range of environmental aspects. For the expert team in particular, our EPF contained various criteria for which they had not previously recognized the relevance for adaptation innovations, and the joint assessment initiated discussions on potential impacts on different time and spatial scales. Furthermore, the application of our EPF helped the experts to realize that NBS could also have negative effects on the environment, likewise grey infrastructural constructions. Like the project leaders, they saw our EPF primarily as a valuable tool to discuss the possible effects of adaptation innovations in a systematic and holistic manner, and to identify potential issues for further *ex*-*ante* exploration, quantitative assessment, research or monitoring.

Because both pilots were planned in the Wadden Sea, which is appointed as Natura2000 site, there was a legal requirement for an *ex*-*ante* appropriate assessment (Dutch legal terminology: ‘*passende beoordeling’*). Although such a *passende beoordeling* discusses specific criteria in great detail (e.g. the abundance of specific species), both project leaders mentioned that our EPF presented additional criteria, that in hindsight could have helped to galvanize the discussion and to increase support, and identify additional issues worthwhile to measure and monitor.

Interestingly, the Mud Motor pilot revealed that the technical design had not correctly accounted for the complex hydrodynamic processes that determine both the transport of the mud and expansion of the salt-marsh. The disposal of large volumes of mud in the nearby tidal channel did not render in the foreseen increase in development of salt-marsh foreland and co-benefits for nature (Baptist et al. 2019).

The Wide Green Dike and the related clay mining project cover different environmental issues. The Wide Green Dike will improve the quality of the coastal landscape, especially when compared with a traditional dike-reinforcement with asphalt and stone revetment on the seaward slope. However, the Wide Green Dike and the excavation pond will cost some of the current salt-marsh foreshore. Therefore, sustainability and the potential contribution to the water quality by removing the surplus of sediment are emphasized. By the application of local mined material, CO_2_-emission through transportation will be avoided. All these topics are covered by the criteria in our EPF, and according to the project leader our EPF is a useful tool to sketch a holistic picture of all benefits and trade-offs of complex adaptation innovations.

### Reflection on the value of the EPF in the development and implementation of adaptation innovations

All consulted EIA experts confirmed the value of our EPF to explicitly draw attention to the environmental impact of structural adaptation innovations to avoid environmental trade-offs, whether or not the innovations are mandatory to an EIA. Both the expert from the consultancy firm and the expert from the dredging company raised that in the current situation only final designs are compared in an EIA, but developers of innovations want to be able to make adjustments during the design process and appreciate advice on how they can take the environment into account. According to these experts, this is especially true for nature-based adaptations, where designers are interested to explicitly include natural processes and to know upfront how and where to include room for adjustments and compensation for environmental trade-offs. For example, to approve the implementation of the Mud Motor, it was necessary to prove that the design included natural processes to create new habitats.

In the discussions also emerged that specifying sustainability and emphasizing the long-term perspective of environmental co-benefits is helpful to arrange funding and to galvanize the implementation process of innovations. The design and implementation of the Wide Green Dike, for example, proved to be a very challenging process. The explicit connection with environmental policies and pending tasks, such as the legal obligation to improve the water quality in the Ems estuary and the creation of new habitats, ensured that a wide range of stakeholders was willing to cooperate and to overcome concerns about local and temporary disturbance of habitats. According to the EIA experts, designers are thus interested in advices on mainstreaming and connecting with related policies.

Furthermore, it was raised that normally the regional authority in collaboration with the national EIA commission decides if an innovation is mandatory to an EIA, and moreover, formulates and checks the issues covered by the EIA. According to the expert from the regional authority, this includes actual and tailored environmental topics, which are also covered by the broad range of sustainability-related criteria in our EPF. The national EIA expert observes in EIAs an increase in attention for issues related to adaptation strategy and adaptability, and sees merits for guidance on the environmental impacts of adaptation innovations in the steps preceding an EIA.

The EIA experts advised to make the EPF tool available via national websites that provide support in the development of adaptation strategies and measures (e.g. www.klimaatadaptatienederland.nl). They advised to stimulate a broad adoption by providing workshops for adaptation professionals and developing training material for higher education. Furthermore, a role for consultancy firms was identified in guidance and the follow up of the self-assessment results.

## Discussion

### Selected evaluation criteria

We developed an EPF that pragmatically integrates and operationalizes various sustainability-related concepts and approaches. It contains indicators for water, soil and air and for nature based on environmental related agreements and legislation, experiences with existing evaluation frameworks and literature on the wide range on indicators. When applied in case studies our EPF highlighted several criteria previously not recognized by the experts as relevant for climate adaptations, and helped them to get a more comprehensive picture of potential environmental impacts and of connections with other relevant approaches and policy fields. They experienced that our EPF can facilitate discussion through its clear questions that urge to explicitly describe environmental impacts, and help to identify important knowledge gaps regarding environmental co-benefits and trade-offs. Precisely for innovative measures, with which by definition little experience has been gained (Enríquez-de-Salamanca et al. 2017), this may be helpful for discussions between stakeholders with different interests and to identify possible environmental trade-offs upfront.

Our Wide Green Dike case study illustrates that *ex*-*ante* impact assessment of innovations can help to highlight knowledge gaps and differences between stakeholders in insights, and might help to overcome the innovation implementation gap by providing targeted information or developing a joint learning process (Schmid et al. 2016; Bellinson and Chu 2018; Wamsler et al. 2020). The Wide Green Dike monitoring programme will ultimately result in more insights about the complex accretion and seepage processes, trade-offs, and whether compensation or additional measures are required. However, we would like to point out that for a joint and balanced learning process, targeted to overcome implementation barriers, also the innovation’s technical (Lendering et al. 2018), social (Bellamy 2019), and sectoral performance needs to be taken in account (see Fig. 2).

Although the innovators and end-users that were iteratively consulted during the development of our framework had a strong preference for a (qualitative) method that they could initially apply themselves, our real-world case studies illustrated that quantitative data may be needed to overcome implementation gaps. For some criteria such quantitative data may be available and accessible via databases or could be obtained via scenario analysis or modelling. However, then also insight in spatial and temporal scales is important to compare the adaptation innovation’s impact with the reference situation and other measures (see e.g. Baker et al. 2013; Pan et al. 2018; Turkelboom et al. 2018), which is not trivial due to feedbacks in the complex human-ecological systems.

### Benefits of a comprehensive and holistic assessment

Our study is limited to two adaptation innovations. Nevertheless, it illustrates convincingly that systematic and holistic environmental assessment can reveal important insights on environmental co-benefits and trade-offs that otherwise might have been overlooked. For instance, impacts of the Mud Motor like greenhouse gas emission and influence on saline seepage in the hinterland were upfront (in the legally required detailed assessment) not considered but later turned out to be key in the *ex*-*post* evaluation. Such discrepancies in environmental assessments were also mentioned by Kørnøv and Wejs (2013), who have observed that despite legal requirements, local authorities have some freedom to determine whether the application of environmental assessments is mandatory, or whether the likely impacts are significant or not. While more research would be needed to assess the impact of such discretion in relation to potential environmental impacts, Kørnøv and Wejs (2013) found this can lead to significant gaps in regulation of climate change related mitigation and adaptation innovations (Larsen, Kørnøv and Wejs 2012; Wende et al. 2012).

In addition, our study confirms the growing realization that NBS, like other adaptation innovations, may have environmental trade-offs in addition to their intended performance and co-benefits (van Loon-Steensma and Vellinga 2013). This is in line with conclusions by Raymond et al. (2017) in their large study of urban NBS that one can expect environmental costs as well as benefits of NBS, and that all of these effects should be considered in the development and implementation of these measures. Simply labelling an innovation as ‘nature based’ does not guarantee it will be effective or environmentally sensitive.

Existing discussions about NBS tend to focus on the application of relatively small-scale solutions applied within the urban environment (Scott and Lennon 2016; Raymond et al. 2017; Cariñanos et al. 2017; Van der Jagt et al. 2017). Such NBS are mostly limited to the interface between green and grey infrastructures, and on the regeneration of neglected urban spaces, as a way to mitigate the impact of an increase in flooding and heat within the urban environment, and to restore degraded ecosystems in urbanized areas (EC 2015). While such innovations do not preclude considerations of nature conservation and development, the potential impact is limited in that they are implemented in an already built environment. Large-scale nature-based solutions implemented within natural environments (e.g. Building with Nature solutions like the Dutch Sand-Motor, and our case study adaptations), on the other hand, have a very different relationship with nature conservation. The early application of the EPF in the development of adaptation innovations, as we argue for in this paper, is designed to pre-empt some of the unintended or overlooked environmental consequences at the design and pilot stages as to enable adaptation prior to socio-technical lock in and the production of uncomfortable knowledge (Rayner 2012). Rayner explores a particular kind of uncertainty, in which information is omitted or not sought as a way to maintain a particular environmental policy agenda. While we would not claim this to be the case in relation to the development and political prioritization of NBS, we do claim that the application of the EPF with multiple and varied stakeholders can yield diverse perspectives that can reduce the possibility of unintended and unpredicted environmental consequences.

### Value of the EPF for the implementation of innovations

In the literature, the importance of connecting, complementing and mainstreaming climate adaptation strategies and measures with other policies is generally recognized in order to gain support and to overcome legal preconditions and other obstacles to actually take innovations further (e.g. Biesbroek et al. 2013; Nalau, Becken and Mackey 2018; Runhaar et al. 2018). Our case studies revealed that it was crucial to stress and underpin all potential co-benefits of a (temporal) shift in habitats for nature and the environment in a long-term perspective. Interestingly, it is precisely the connection of NBS with policies on sustainability and biodiversity conservation that is strongly emphasized in scientific literature and policy documents (e.g. EC 2015; Cohen-Shacham et al. 2016; Kabisch et al. 2016; Faivre et al. 2017), while it does not transcend conceptual approaches (Narayan et al. 2016; Nature 2017; Seddon et al. 2020). Our EPF encompass criteria that pragmatically operationalize approaches like sustainability, green economy, circular economy, ecosystem services and policies on environmental quality and protection (including biodiversity) to help innovators and end-users (including licensing authorities) to make these connections.

In the current practice of implementing adaptations, only final designs of adaptation innovations with foreseen environmental impacts are discussed and compared in an EIA. While it would be especially helpful during the design process to gain insight in possible environmental trade-offs and to receive guidance on how to take the environment into account and where to include room for adjustments and compensation for environmental trade-offs. Given the increasing need for sustainable measures, a tool such as our EPF should be easily accessible for innovators—e.g. through national websites—to provide guidance on the environmental impacts of climate adaptation innovations in the steps before an EIA. Moreover, explicit attention to the environmental impact of all structural adaptation innovations, regardless of whether the innovations are mandatory to an EIA, could avoid environmental trade-offs, and help to transform towards sustainable development (Smith et al. 2010).

### Reflection on the study’s limitations

Although our EPF adopts an interdisciplinary approach, it is difficult to cover all relevant approaches and encompass all viewpoints, and moreover, to translate this into clear and meaningful criteria. Furthermore, several indicators are sensitive to different geographical contexts. Therefore, future research should explore how to tailor the EPF to different contexts.

An important limitation of our framework is that not all criteria can be easily quantified or spatially delineated, while spatial explicitness allows a systematically comparison of the innovation’s impact with the reference situation and other measures. Furthermore, spatial explicitness offers an avenue to connect our framework with information and databases on nature (e.g. www.natura2000.eea.europe.eu), related approaches (e.g. Mapping and Assessment of Ecosystems and their Services (EC 2013b)) and relevant models (e.g. land-use models, see e.g. Baker et al. 2013; Pan et al. 2018; Turkelboom et al. 2018).

Our choice to pilot test our framework on two currently implemented adaptation innovations has methodological limitations, because our framework is meant as a tool in the design process and to overcome implementation hurdles. Although the assessment by uninvolved graduate students formed a proxy for *ex*-*ante* assessment of real-world nature-based adaptation innovations, the application of the EPF formed rather an *ex*-*post* assessment. Therefore, application of our EPF early in the design process of (nature-based) adaptation innovations will render in more insights about the value of our EPF.

Despite these limitations, we feel our framework forms an important contribution to identifying the actual co-benefits and trade-offs of adaptation innovations and gearing the design accordingly.

## Conclusion

We developed an Environmental Performance Framework (EPF) to assess adaptation innovations. This integrated self-assessment tool was based on input and feedback from climate adaptation innovators and end-users, literature on environmental performance in the context of adaptation policy, and on experiences with adaptation assessment frameworks. The EPF is indicator-based and includes criteria on (1) the sustainability of the adaptation’s design and its contribution to sustainable development, (2) the quality of the environment, and (3) the quality of ecological systems. The EPF has been found adequate during initial testing.

Our study demonstrates that the EPF forms a pragmatic tool to discuss and assess the environmental effects of adaptation innovations *ex*-*ante* in a systematic and holistic way, and connect climate adaptation innovations to sustainability and related policy fields. Such a connection with other policies and a long-term perspective on environmental co-benefits proved crucial for our two NBS adaptations to overcome implementation hurdles. We also found that, to address knowledge gaps or to bridge differences in knowledge between stakeholders, a comprehensive set of criteria helps to identify environmental issues worthwhile to measure and monitor.

Our study furthermore revealed that there is an interest in guidance during the design process of adaptation innovations on how to take the environment into account, on how to integrate natural processes into the design, and on how to deal with room for adjustments and compensation for environmental trade-offs. Availability of the EPF via e.g. websites can support designers to optimize the design on environmental co-benefit from the outset, and prevent that environmental issues emerge through an EIA of the final design.

Application of our EPS on the two large-scale NBS adaptation innovations pinpointed several environmental issues, like an impact on the fresh water availability in the hinterland and a temporal change in habitats. Our systematic and comprehensive framework can aid to explicitly consider such unforeseen environmental impacts of NBS upfront. We therefore argue for the explicit consideration of environmental impact in the design process of all climate adaptation innovations, including NBS, in addition to technical performance and costs.

Future work would be needed to further explore the value of our framework in the design and the facilitation of the implementation process of adaptation innovations.
